# Identification of the Key Genes of Autism Spectrum Disorder Through Protein-Protein Interaction Network

**DOI:** 10.31661/gmj.v0i0.1367

**Published:** 2019-05-11

**Authors:** Mona Zamanian Azodi, Mostafa Rezaei Tavirani, Majid Rezaei Tavirani

**Affiliations:** ^1^Proteomics Research Center, Shahid Beheshti University of Medical Sciences, Tehran, Iran; ^2^Proteomics Research Center, Faculty of Paramedical Sciences, Shahid Beheshti University of Medical Sciences, Tehran, Iran; ^3^Faculty of Medicine, Iran University of Medical Sciences, Tehran, Iran

**Keywords:** Autism Spectrum Disorder, Transcriptome, Protein Interaction Maps, Gene Ontology

## Abstract

**Background:**

Currently, the prevalence of autism spectrum disorder (ASD) is increasing, which widely spurs the interest in the molecular investigation. Thereby, a better understanding of the given disorder mechanisms is likely to be achieved. Bioinformatics suiting protein-protein interactions analysis via the application of high-throughput studies, such as protein array, is one of these achievements.

**Materials and Methods:**

The gene expression data from Gene Expression Omnibus (GEO) database were downloaded, and the expression profile of patients with developmental delay and autistic features were analyzed via Cytoscape and its relevant plug-ins.

**Results:**

Our findings indicated that EGFR, ACTB, RHOA, CALM1, MAPK1, and JUN genes as the hub-bottlenecks and their related terms could be important in ASD risk. In other words, any expression modification in these genes could trigger dysfunctions in the corresponding biological processes.

**Conclusion:**

We suggest that differentially expressed genes could be used as suitable targets for ASD after being validated.

## Introduction

Autism spectrum disorder (ASD) as a prevalent neurodevelopmental condition is on the rise with the rate of 1/68 to 1/50 persons [1]. The typical ASD symptoms approved by Diagnostic and Statistical Manual of Mental Disorders, Fifth Edition (DSM-5), include social communication/interaction disability, repetitive behaviors, and sensory impairments [2]. Genetic and environmental factors have also a considerable role in this heterologous disorder [3]. Many related etiological factors such as mitochondrial dysfunction [4], heart rate [5], Zn/Cu levels [6], and serotonin system [7], have been suggested for the given disorder. Behavioral evaluations are the only diagnosis methods for this complex disorder [8]. Identification of the molecular signatures could also help understand the pathophysiological mechanisms and consequently improve the diagnosis and treatment approaches of ASD [1]. In this light, many biomarker investigations of this heritable disorder have been widely studied via genomics, proteomics, and metabolomics [1, 8]. However, identification of reliable biomarkers still requires more studies [9]. Genetic evaluations carried out on this concept introduced *SHANK3* mutation as one of the key risk factors of ASD [10]. Gene expression profiling is one of the ways to determine the gene expression changes in specific conditions such as neurological disorders [11]. Also, bioinformatics are another relatively new discipline that can suggest other aspects of biomolecules identified from high-throughput studies. In fact, a modified condition, such as a disease state, is responded by the functionalization resulted from the interactions between molecules [12]. Some elements are more crucial in this regard due to their central roles in a protein-protein interaction (PPI) network [13]. Any disruption in these essential nodes could trigger abnormal conditions such as a disease. Thus, detecting significantly differentially expressed genes (DEGs) with such a feature could add more reliability to their purposed candidacy for diseases such as ASD. For this aim, the present study was designed to provide further insight into the DEGs in ASD via the PPI network analysis.

## Materials and Methods

There are many genes related to ASD, which can be screened to find the critical ones. In this study, the genes associated with the autistic patients with dysregulated mood compared to the healthy individuals were extracted from the Gene Expression Omnibus (GEO) database and analyzed via bioinformatics.

### Data Collection

Gene expression data related to the patients with global developmental delay and autistic features and healthy individuals were downloaded from the GEO database. The dataset entitled “expression data from patients with global developmental delay and autistic features and normal controls” with accession number GSE29691 and platform GPL570 was selected to this end.

### Statistical Analysis

DEGs were assigned and analyzed using the GEO2R online software,GEO, https://www.ncbi.nlm.nih.gov/geo. Prior to DEGs analysis, the groups of samples were first compared via boxplot to assess the quality of gene expression data and ensure whether the samples are comparable in this regard. The next step was to determine these DEGs and assign the related statistical properties. Among the top 250 significantly expressed genes, those with 0.5 ≥ fold change (FC) ≥ 1.5 and adjusted P-value less than 0.05 were considered for further analysis.

### PPI Analysis

The selected genes with gene name were categorized as up- and down-regulated ones, and then queried in Cytoscape, a PPI network analyzer [14]. Moreover, STRING database was the platform for retrieving a network of interacting genes. This application is available in Cytoscape with four sources including STRING protein, STITCH, DISEASES, and PubMed [15]. Edge score and number of maximum additional interactions were assigned as 0.4 and 50 for the network construction. The network was analyzed further, and the centrality features were assessed by the Network Analyzer application based on two important parameters including degree centrality (DC) and betweenness centrality (BC). Nodes with the highest degree and betweenness values are called hub-bottlenecks [16]. The hub-bottleneck nodes were selected for expression analysis via CluePedia query and merged with GSE29691 expression data file. More focus was on genes with significant expression values in the dataset. CluePedia application could provide enrichment analysis for genes, proteins, and miRNAs by considering linear and non-linear statistical dependencies [17]. Furthermore, the enrichment analysis of these genes was carried out by STRING Enrichment analysis with P≤0.05. For this aim, at first, a sub-network of the most significantly differentially expressed hub-bottlenecks was constructed by STRING Plug-in, and then the gene ontology was assigned to each.

## Results

**Figure 1:**
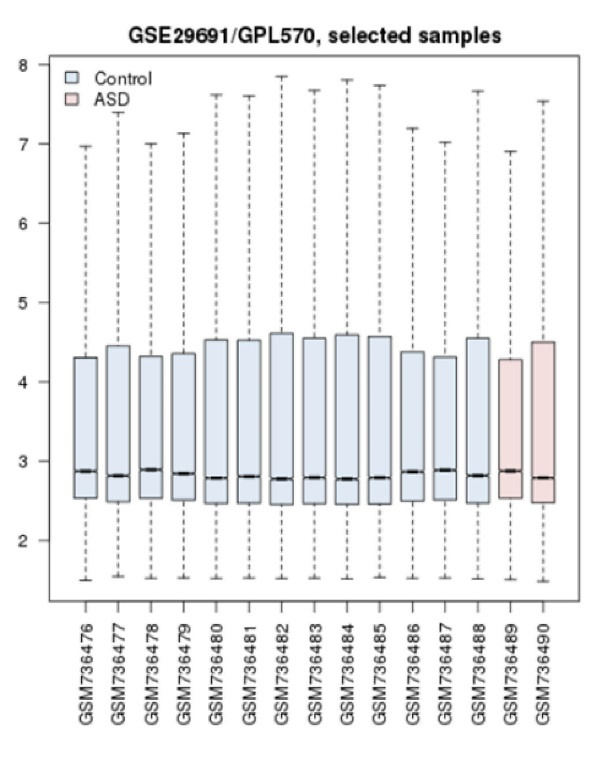
The boxplot comparison of the groups of samples (2 healthy and 13 ASD). The healthy group is assigned a pink color and disorder group is assigned a blue color.

**Figure 2:**
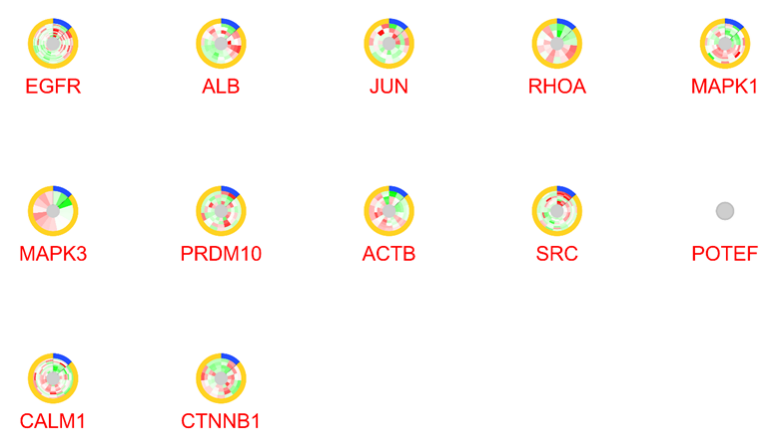
The normalized expression values of all genes that show some expression data with different spots. The data were extracted from GSE29691 through CluePedia. No expression data was available for POTEF. Healthy samples and ASD patients are shown by yellow and blue, respectively. Color scheme change from red to green indicates the changes in the expression values from the maximum positive to negative expression (white=zero expression and grey=missing values).

**Figure 3:**
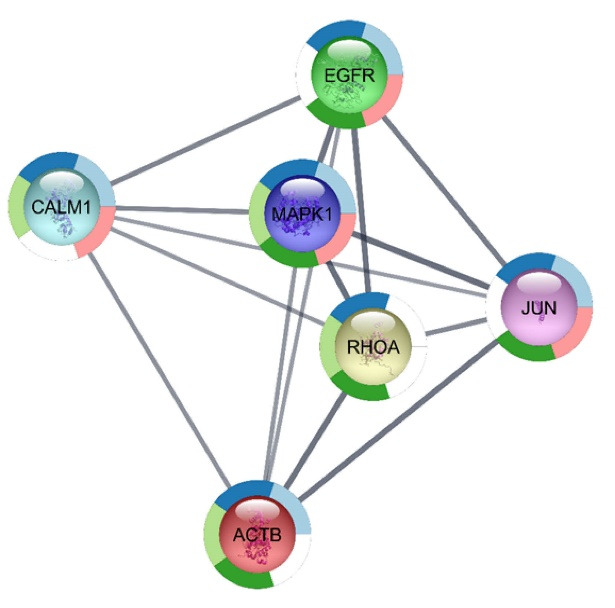
The functional enrichment analysis of the six significantly differentially expressed hub-bottlenecks with 15 interactions among them. Different colored parts of the circles refer to the related biological processes. P≤ 0.05 was considered as the significant level.

Overall, 13 ASD samples and 2 healthy ones were compared in terms of expression values. As shown in Figure-1, data from box-plot analysis indicated that the samples are median-centered and are qualified to continue for more analysis. The expression comparison indicated that there are genes with differential expressions. These genes are ranked based on adjusted p-value. Among the top 250 genes with adjusted P< 0.05, the genes with 0.5 ≥ FC ≥ 1.5 were identified to be included in the PPI network. A PPI network was constructed with these properties: 98 nodes and 1028 links. In this network, genes with high values of centralities were determined via Network Analyzer, and 12 common genes (as hub-bottlenecks) of 20% of top ones (highest BC and DC values) are listed in Table-1.To evaluate the hub-bottlenecks expression profile, the genes were then queried via CluePedia panel and merged with GEO expression data as shown in Figure-2. Genes with at least one significantly differential expression value are listed in Table-2. The knowledge obtained through searching gene expression data values from all genes indicates that except for *CALM1*, there was one significant expression value for *EGFR*, *JUN*, *RHOA*, *MAPK1*, and *ACTB*. There are four differentially expressed spots for *CALM1*, which the most significant ones are included in Table-2. To acquire more information about the six significantly differentially expressed hub-bottlenecks, namely *CALM1*, *EGFR1*, *MAPK1*, *ACTB*, *RHOA*, and *JUN* the functional analysis of them via STRING Enrichment application was carried out, and the most significant ones were assigned specific colors. The top five biological processes were chosen to this end (Figure-3 and Table-3).

## Discussion

The etiology of ASD has remained unknown; however, molecular biology examination has proved to be promising in different kinds of neurological disorders [18, 19]. Here, the gene expression profile of patients with ASD has been compared with that of the healthy ones with the focus on the interaction network decoding. At first, DEGs were derived from the top 250 significantly expressed genes, and then a network of them was constructed. In this network, there were genes with differential properties called hub-bottlenecks. Following the analysis of these genes via designated statistical criteria, a list of genes was introduced. Overall, 12 common genes were obtained that none of them belonged to the top 250 up- and down-regulated genes. The highest degree and betweenness values were obtained for *EGFR* (51 and 0.06, respectively). *JUN* was found to have the lowest degree and betweenness values (39 and 0.02, respectively). In general, 6 out of 12 central nodes had differential expression values in which there are four negative and two positive expressions. *CALM1* was the gene with four significantly differentially expressed spots while other genes were represented with only one significantly differential expression. All the spots in *CALM1* were negatively expressed in ASD. Some of these genes, namely *EGFR*, *ACTB*, *RHOA*, *CALM1*, *MAPK1*, and *JUN* are common in different kinds of diseases [20-27]. All of these genes are reported for cancer pathophysiology. Among them, *EGFR* as the top hub-bottleneck has been widely reported in ASD [15, 28]. However, in Table-2, the expression value and the statistical properties of the sated gene are presented among the other significantly differential expressions. Development and repair of the nerve cell is the responsibility of this molecule. In line with the previously reported results [28, 29], the present study also revealed that the given gene has a positive expression profile in ASD. *ACTB* as a cytoskeletal protein had a negative expression in ASD, as previously showed the same manner in developmental abnormalities [30]. The changes in this gene can disrupt the functions of some organs including brain, heart, and kidney [31]. *RHOA*, as a participant in neural development [32], implies ASD [33]. The down-regulation of this gene was observed, and its centrality can suggest the more fundamental roles of this candidate. The next hub-bottleneck is *CALM1* with four significant reduced amounts of expression in ASD. No particular relationship between this gene and autism has been reported so far. *MAPK1*, as a member of the *MAPK* family, has an essential role in proliferation and apoptosis. Indeed, this gene is a potential biomarker in cancer [34]. What is more, apoptosis is one of the contributing mechanisms in ASD [35]. Therefore, it may cause impairment in neurological development, resulting in many neurodevelopmental disorders [36, 37]. One of the features of ASD is an impairment in social communications. Apparently, this gene is implicated in this phenotype in the central nervous system as suggested by some investigations [38]. Also, *JUN* is important in cell survival and apoptotic activities [39]. No data is available about the relationship between this gene and ASD. However, the role of this gene in apoptosis could justify its putative effect on ASD. In other words, *MAPK1* and *JUN*, as mentioned earlier, are active in apoptosis that may be related to the pathogenesis feature of the ASD. Further examination of these central hub-bottlenecks showed that there are five highlighted biological processes for a network of these essential genes. Our results indicated that at least four of these genes contributed to an important biological process. Among these terms, the FC receptor signaling pathway is the main one. *MAPK1* is the gene contributing to all the important biological processes. By expression modifications in these genes, the related biological processes may be influenced. In other words, each of these terms may lose their function by differential expression of these hub-bottlenecks. On the whole, some of these genes, such as *EGFR*, were previously shown as promising candidates for ASD compared to others, e.g., *JUN*. It merely confirms both categories regarding centrality aspect in a PPI network. Therefore, the linkage of *EGFR*, *ACTB*, *RHOA*, *CALM1*, *MAPK1*, *JUN*, and their associated biological processes with ASD based on the PPI network analysis is supported.

## Conclusion

The differentially expressed hub-bottlenecks and biological terms might be relevant targets for the improvement of ASD.

## Acknowledgment

The authors are deeply grateful for the support of Proteomics Research Center of Shahid Beheshti University of Medical Sciences

## Conflict of Interest

There is no any conflict of interest.

**Table 1:** The List of Hub-Bottlenecks Including Genes with the Highest BC and DC Values

**Genes**	**DC**	**BC**
**EGFR**	51	0.06
**MAPK1**	51	0.03
**SRC**	49	0.02
**PRDM10**	48	0.04
**MAPK3**	47	0.02
**CALM1**	47	0.03
**ACTB**	46	0.02
**RHOA**	44	0.02
**POTEF**	41	0.03
**CTNNB1**	41	0.03
**ALB**	40	0.03
**JUN**	39	0.02

**DC:** degree centrality; **BC:** betweenness centrality

**Table 2:** The List of Hub-Bottlenecks with Significantly Differential Expression Values and Their Properties Including Expression Type, FC, and Significance

**Genes**	**Expression type**	**FC**	**P-value**
***CALM1***	Negative	H 2.09	L 1.87e-04
***EGFR***	Positive	1.69	6.36e-03
***RHOA***	Negative	1.62	3.25e-02
***ACTB***	Negative	2.09	6.57e-03
***JUN***	Positive	1.76	3.40e-04
***MAPK1***	Negative	1.54	2.49e-02

**Table 3:** The List of Biological Processes Related to the Six Hub-Bottlenecks and Their Significant Contributing Genes with Their Assigned False Discovery Rate (FDR) P-value.

**Description**	**Color in model**	**Enriched genes**	**FDR P-value**
FC receptor signaling pathway	Light blue	*MAPK1|EGFR|CALM1|ACTB|JUN*	1.13E-05
enzyme-linked receptor protein signaling pathway	Dark blue	*MAPK1|EGFR|CALM1|ACTB|JUN|RHOA*	3.61E-05
vascular endothelial growth factor receptor signaling pathway	Light green	*MAPK1|CALM1|ACTB|RHOA*	5.22E-05
axon development	Dark green	*MAPK1|EGFR|ACTB|JUN|RHOA*	1.37E-04
Fc-epsilon receptor signaling pathway	Pink	*MAPK1|EGFR|CALM1|JUN*	1.71E-04
